# Accuracy of optical diagnosis with narrow band imaging in the surveillance of ulcerative colitis: a prospective study comparing Kudo, Kudo-IBD and NICE classifications

**DOI:** 10.1007/s00384-024-04635-6

**Published:** 2024-05-24

**Authors:** Andrea Cassinotti, Piergiorgio Duca, Giovanni Maconi, Elena Beretta, Gianluca Matteo Sampietro, Alessandro Pellegrinelli, Manuela Nebuloni, Sandro Ardizzone

**Affiliations:** 1 Gastroenterology Unit, ASST Fatebenefratelli Sacco, Luigi Sacco University Hospital, Milan, Italy; 2 Gastroenterology and Digestive Endoscopy Unit, ASST Sette Laghi, Varese, Italy; 3 Chair of Statistics, University of Milan, Milan, Italy; 4 Department of Biochemical and Clinical Sciences “L. Sacco”, University of Milan, Milan, Italy; 5 Division of General and HPB Surgery, ASST Rhodense, Rho Memorial Hospital, Rho, Milan, Italy; 6 Pathology Unit, ASST Fatebenefratelli Sacco, Luigi Sacco University Hospital, Milan, Italy

**Keywords:** Chromoendoscopy, Kudo, Kudo-IBD, NBI, NICE, Ulcerative colitis

## Abstract

**Purpose:**

The diagnostic accuracy of Narrow Band Imaging (NBI) in the endoscopic surveillance of ulcerative colitis (UC) has been disappointing in most trials which used the Kudo classification. We aim to compare the performance of NBI in the lesion characterization of UC, when applied according to three different classifications (NICE, Kudo, Kudo-IBD).

**Methods:**

In a prospective, real-life study, all visible lesions found during consecutive surveillance colonoscopies with NBI (Exera-II CV-180) for UC were classified as suspected or non-suspected for neoplasia according to the NICE, Kudo and Kudo-IBD criteria. The sensitivity (SE), specificity (SP), positive (+LR) and negative (-LR) likelihood ratios of the three classifications were calculated, using histology as the reference standard.

**Results:**

394 lesions (mean size 6 mm, range 2–40 mm) from 84 patients were analysed. Twenty-one neoplastic (5%), 49 hyperplastic (12%), and 324 inflammatory (82%) lesions were found. The diagnostic accuracy of the NICE, Kudo and Kudo-IBD classifications were, respectively: SE 76%-71%-86%; SP 55-69%-79% (*p* < 0.05 Kudo-IBD vs. both Kudo and NICE); +LR 1.69-2.34-4.15 (*p* < 0.05 Kudo-IBD vs. both Kudo and NICE); -LR 0.43-0.41-0.18.

**Conclusion:**

The diagnostic accuracy of NBI in the differentiation of neoplastic and non-neoplastic lesions in UC is low if used with conventional classifications of the general population, but it is significantly better with the modified Kudo classification specific for UC.

## Introduction

Patients with long-standing ulcerative colitis (UC) are at increased risk of colorectal cancer (CRC) [1]. Periodic colonoscopies are, therefore, recommended by international guidelines on inflammatory bowel disease (IBD) [2].

Multiple endoscopic strategies and technologies for high-quality colonoscopy have been analysed for both detection and characterization of polypoid and non-polypoid lesions in IBD. However, while both dye-based chromoendoscopy (DCE) and virtual chromoendoscopy (VCE) have been recently included as the preferred strategies for detection of neoplasia in IBD [3], no methods are still clearly accepted for the differentiation between neoplastic and non-neoplastic lesions in IBD (the so called “optical diagnosis”), due to controversial data on their accuracy. This is an important limitation, because non-neoplastic lesions, especially inflammatory lesions, are the most frequent visible lesions in IBD and share similar macroscopic morphology and size to neoplasia [4–8].

Recent studies have questioned whether the unsatisfactory accuracy of current methods of lesion characterization is an inherent feature of the technological imaging processing, or is influenced by the criteria of lesions classification which were used in previous studies [9, 10]. For example, Narrow Band Imaging (NBI) has been previously associated to high accuracy in optical diagnosis during CRC screening of average risk subjects [11], but failed to show its superiority in early studies on both dysplasia detection and characterization in IBD [12]. Notably, all these studies used the conventional Kudo classification of mucosal pit-patterns [13–15], which is widely used for optical diagnosis in the screening of general population [16], but showed unsatisfactory accuracy in UC [7, 8]. Only a recent multicenter study achieved a high negative predictive value of 88% for Kudo’s classes I and II (usually considered not suspected for neoplasia) [17], which is near to the 90% threshold recommended by ASGE for clinical utility in the context of lesion characterization [18].

More recently, two studies using VCE with Fuji Intelligent Color Enhancement (FICE) described better diagnostic accuracy of a modified classification of Kudo, specific for UC, by adding three endoscopic markers (fibrin cap, pits heterogeneity and vascular intensity) as modifiers of the neoplastic risk [7, 8]. Another endoscopic classification, named NBI International Colorectal Endoscopic (NICE) classification, is based not only on surface pits criteria but also on color and vascular pattern; it has been validated for use in CRC screening of the general population, with a pooled sensitivity of 98% and 95% negative predictive value for lesion characterization [19, 20]. However, no studies have been performed with the NICE system in IBD.

In this prospective study, we compare the diagnostic accuracy of conventional Kudo and NICE classifications with the modified Kudo classification for IBD (Kudo-IBD), in the characterization of lesions during surveillance endoscopy for UC.

## Materials and methods

### Study design

This was a prospective study on consecutive patients with at least one polypoid or non-polypoid lesion detected during surveillance colonoscopy with NBI for long-standing UC.

Three endoscopic classifications of mucosal patterns were used to predict in vivo the nature (neoplastic vs. non-neoplastic) of each lesion. Their diagnostic accuracy on optical diagnosis was calculated, using histology as reference test.

The study was performed in accordance with the Declaration of Helsinki and its later amendments. It was approved by the local Ethical Committee, and patients signed an informed consent form to undergo endoscopic examination.

### Inclusion and exclusion criteria

The inclusion criteria were a previous diagnosis of UC according to international guidelines, disease duration ≥ 8 years since onset of symptoms, and at least one visible, polypoid or non-polypoid, lesion of any size and morphology, detected during surveillance colonoscopy. Any clinical and endoscopic activity was permitted, according to the Mayo score, in order to test the three classifications in the common clinical real life.

Exclusion criteria were non-correctable coagulopathy, melanosis coli, hereditary polypoid syndromes, and poor bowel preparation (Boston Bowel score < 2 in any colorectal segment).

### Endoscopic protocol

Firstly, full colonoscopy with white light was performed up to the caecum; no biopsies or resections were performed during the insertion phase. Retroflexion in the right colon and secon look were performed when considered convenient according to the endoscopist’s opinion. NBI was then activated during extubation along the entire colon, starting from the caecum. The Exera-II CV-180 Olympus colonoscope was used. Any polypoid or non-polypoid lesions found during extubation were recorded and classified in-vivo according to their morphology, size, location, mucosal and vascular pattern, before tissue sampling or resection. Targeted biopsies of lesions not suspected for neoplasia, or full endoscopic resection of lesions suspected for neoplasia, were finally performed. All procedures and classifications of lesions were performed by a single operator with expertise in surveillance endoscopy for IBD (> 500 procedures performed).

### Classification of lesions

The location, size and morphology of any visible lesion were recorded during real-time endoscopy.

Lesion size was estimated in comparision with a fully opened biopsy forceps or polypectomy snare next to the lesion.

The morphology was described in accordance with the Paris classification for macroscopic aspects [21], while the surface pattern was described according to three distinct classifications.

**Fig. 1:**
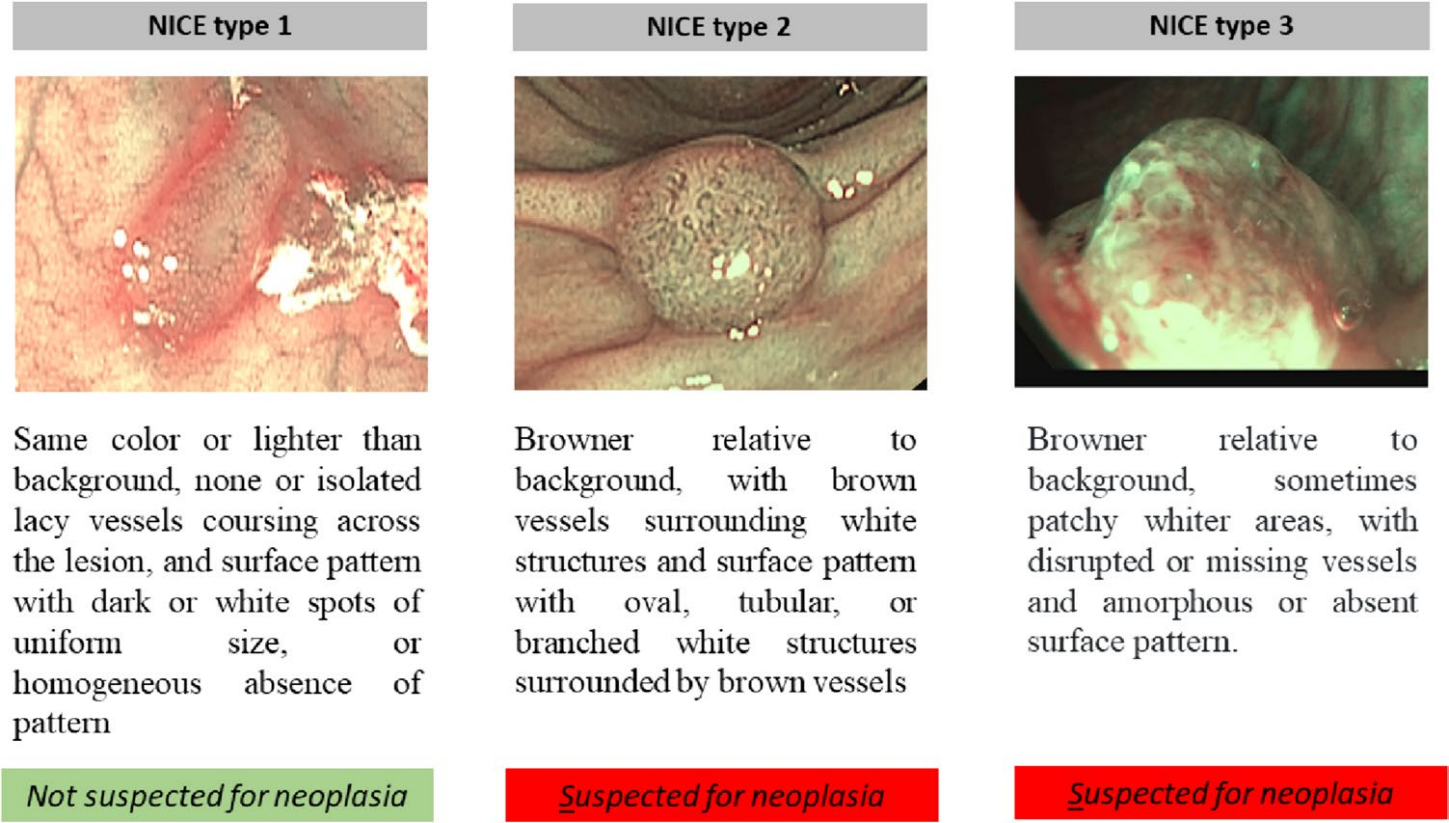
NBI International Colorectal Endoscopic (NICE) classification

The NICE classification (Fig. 1) focuses on color, mucosal and vascular patterns of the lesion: *type-1* lesions were considered not suspected for neoplasia, while *type-2* and *type-3*, respectively associated to adenoma and invasive cancer, were considered suspected for neoplasia [19, 20].

**Fig. 2:**
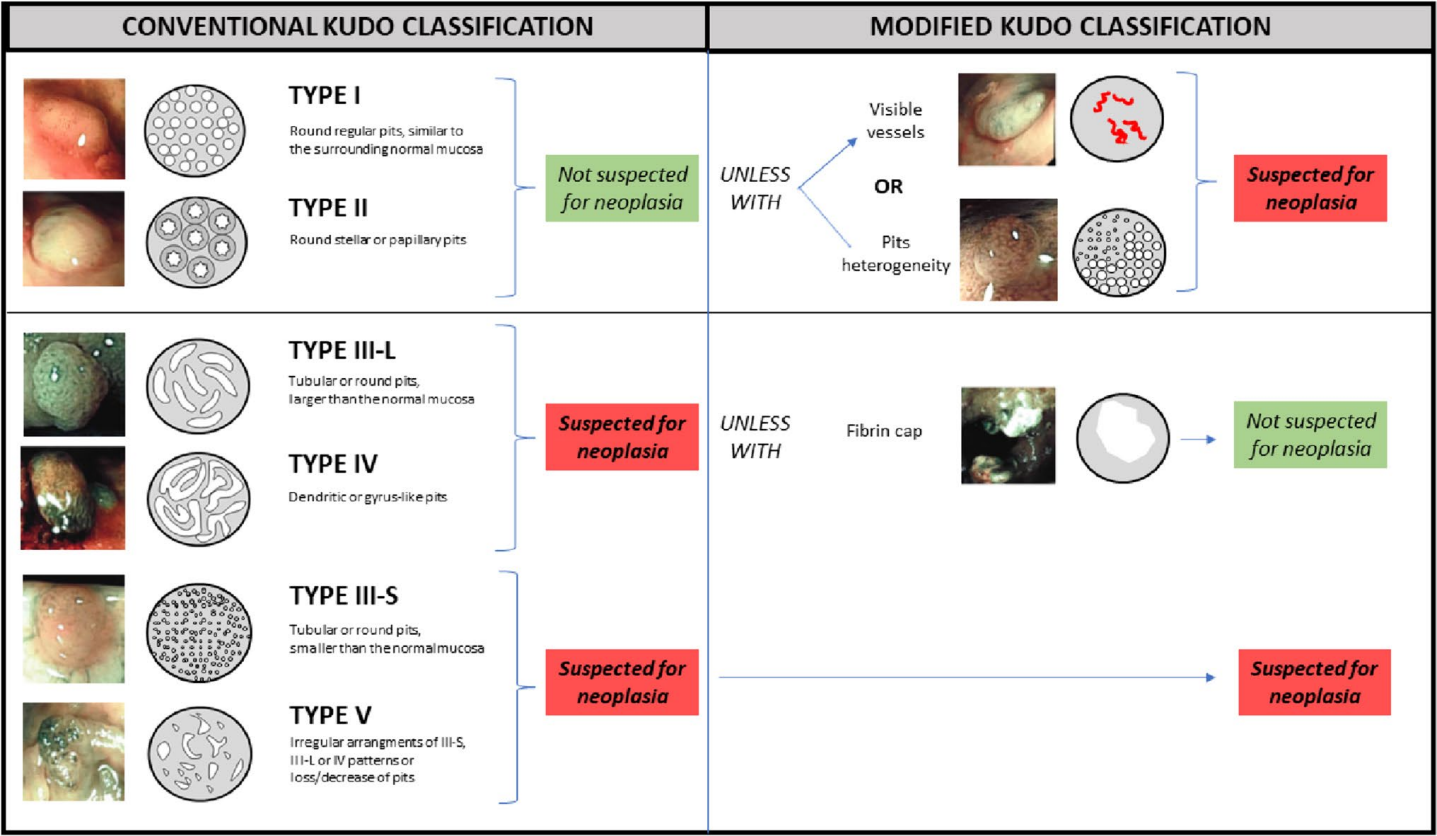
Conventional Kudo classification and modified Kudo classification for IBD (Kudo-IBD).

The conventional Kudo classification analyzes the mucosal pit-pattern (Fig. 2); patterns I and II are not considered suspected for neoplasia, while type III-L, III-S, IV and V are considered suspected for neoplasia [16].

According to the modified classification of Kudo (Kudo-IBD) [7, 8], three specific endoscopic modifiers for risk of neoplasia are added to the original version. In particular, as shown in Fig. 2, Kudo pit-patterns I and II were upgraded as neoplastic lesions if associated with at least one of two further endoscopic factors: (1) irregular, intensive, brown visible vessels, and (2) pits heterogeneity, defined as variable density or size of type I and II pits. In contrast, Kudo pit-patterns III-IV were downgraded to non-neoplastic lesions if associated with a fibrin cap, defined as a non-removable, circumscribed, white exudate covering at least 25 % of the surface of the lesions, which has been associated with inflammatory non-neoplastic lesions in previous studies in IBD [7, 22]. Kudo V and III-S patterns, usually associated with advanced lesions including invasive cancer, were always considered suspected for neoplasia, independently to the presence of the three endoscopic modifiers. Finally, lesions not classified with enough confidence by the endoscopist according to the NICE or conventional Kudo classifications were considered neoplastic *a priori*, but not according to the Kudo-IBD classification if fibrin cap was also detected.

### Histopathological evaluation

Tissue samples were routinely processed and stained. The pathologist was blinded to the endoscopic findings and classified neoplastic and non neoplasic lesions according to current standards [23]. Diagnosis of neoplasia was confirmed by two independent pathologists with expertise in IBD.

### Statistical analysis

Based on previous reports on lesion characterization [10], at least 250 lesions were considered a sample wide enough to have an adequate representation of the various types of histology expected in the real-life, including at leat 5% of neoplastic lesions. Considering that previous characterization studies in UC have described an average of 3–5 polyps per patient [10], at least 80 consecutive patients were required.

Descriptive statistics was used to characterize the study population. Normally distributed data were described by their means (± SD). Categorical variables were compared using the χ^2^ test or the Fisher exact test when appropriate. A 2-sided P-value of ≤ 0.05 was considered significant.

The Standards for Reporting of Diagnostic Accuracy (STARD) guideline was followed in reporting the diagnostic test accuracies of all modalities with respect to lesion differentiation [24].

The primary end-point was to calculate the sensitivity, specificity, positive and negative likelihood ratios of the three classifications applied with NBI, to predict the histology of each visible lesion, using the histologic diagnosis as the reference standard.

Statistical analyses were conducted using IBM-SPSS 21 edition.

## Results

### Patients and lesions

A total of 394 lesions were found in 84 patients. Table 1 summarizes the clinical and endoscopic features of patients and lesions included. The mean number of lesions per patient was 5, with a mean diameter of 6 mm. Macroscopically, most lesions were sessile polyps, followed by slightly elevated non-polypoid lesions. Most lesions were non-neoplastic, in particular 324 inflammatory lesions (82%) and 49 hyperplasic lesions (12%) were found. The remaining lesions (*n* = 21; 5%), from 16 patients (19%) were neoplastic: in particular, 20 low-grade dysplasia and one epithelial proliferation with high-grade dysplasia were detected. In contrast, no invasive adenocarcinomas were found, as well as no sessile serrated lesions, sessile serrated lesions with dysplasia and traditional serrated adenomas. The most frequent location of lesions was the sigma (32%), but neoplastic lesions were more frequently found in the right colon (43%).

**Table 1:** Patients and lesions characteristics

**PATIENTS**	***n*** **(%)**	**LESIONS**	***n*** **(%)**
**Male gender**	52	**Number per patient** **(mean, range)**	5 (1–24)
**Age - years** **(mean, range)**	57 (30–79)	**Size - mm** **(mean, range)**	6 (2–40)
**Disease duration - years** **(mean, range)**	20 (8–48)	**Location – all lesions** *Right colon* *Transverse colon* *Descending colon* *Sigmoid colon* *Rectum*	101 (26%) 56 (14%) 73 (19%) 128 (32%) 36 (9%)
**Primary sclerosing cholangitis**	0 (0%)	**Location – neoplastic lesions** *Right colon* *Transverse colon* *Descending colon* *Sigmoid colon* *Rectum*	9 (43%) 4 (19%) 1 (5%) 5 (24%) 2 (9%)
**Familiar history of colorectal cancer**	20 (24%)	**Macroscopic aspect according to Paris classification** I-s I-p II-a II-b	271 (69%) 5 (1%) 108 (27%) 10 (3%)
**Previous dysplasia**	31 (37%)	**Histology**: *Neoplastic* *Inflammatory* *Hyperplasic polyp* *Invasive cancer*	21 (5%) 324 (82%) 12 (49%) 0 (0%)
**Endoscopic activity**	38 (45%)		

### Diagnostic accuracy of NICE classification

Table 2 shows the distribution of lesions categorized according to the NICE classification. Most lesions were type-1; 2% of them were neoplastic. Type-2 and type-3 lesions were associated with neoplasia in 9% and 17% of cases, respectively. Approximately 8% of lesions were unclassifed, but none were associated with neoplasia.

**Table 2:** Distribution of lesions according to NICE and conventional Kudo classification, and their association with dysplasia

**NICE class**	**No. lesions** **(*n*; %)**	**Dysplasic lesions** **(*n*; %)**
**Type-1**	212 (54%)	5 (2%)
**Type-2**	121 (31%)	11 (9%)
**Type-3**	30 (7%)	5 (17%)
**Unclassified**	31 (8%)	0 (0%)

**Kudo class**	**No. lesions (*n*; %)**	**Dysplasic lesions (*n*; %)**
**Kudo I**	159 (40%)	0 (0%)
**Kudo II**	107 (27%)	6 (6%)
**Kudo III-L**	93 (24%)	14 (15%)
**Kudo III-S**	0 (0%)	0 (0%)
**Kudo IV**	6 (2%)	1 (17%)
**Kudo V**	0 (0%)	0 (0%)
**Unclassified**	29 (8%)	0 (0%)

The sensitivity, specificity, positive- and negative-likelihood ratios of the NICE classification were 76% (95% C.I. 53–92%), 55% (95% C.I. 50–60%), 1.69 (95% C.I. 1.3–2.2) and 0.43 (95% C.I. 0.2–0.94), respectively (Table 3).

**Table 3:** Diagnostic accuracy of the three classifications for lesion characterization in ulcerative colitis

	**NICE**	**Conventional Kudo**	**Kudo-IBD**
**True positives**	16	15	18
**False positives**	168	114	77
**True negatives**	205	259	296
**False negatives**	5	6	3
**Sensitivity**	76% (53–92)	71% (48–89)	86% (64–97)
**Specificity**	55% (50–60)	69% (65–74)	79% (75–83)*
**Positive likelihood ratio**	1.69 (1.3–2.2)	2.34 (1.71–3.19)	4.15 (3.19–5.41)*
**Negative likelihood ratio**	0.43 (0.2–0.94)	0.41 (0.21–0.81)	0.18 (0.06–0.51)

*p ≤ 0.05

### Diagnostic accuracy of conventional Kudo classification

Concerning the conventional Kudo classification, most lesions (67%) had a pit pattern not suspected for neoplasia (type I or II), while no lesions had a pattern III-S or V, in accordance with the absence of invasive lesions in our cohort (Table 2).

While type I was never associated with neoplasia, 6% of lesions classified as type II were neoplastic (all with low-grade dysplasia). In contrast, among conventional type III-L or IV lesions, only 15% were associated with neoplasia. In addition, 29 lesions (8%) were unclassified, but none neoplastic.

The sensitivity, specificity, positive- and negative-likelihood ratios were 71% (95% C.I. 48–89%), 69% (95% C.I. 65–74%), 2.34 (95% C.I. 1.71–3.19) and 0.41 (95% C.I. 0.21–0.81), respectively (Table 3).

### Diagnostic accuracy of modified Kudo classification

**Fig. 3:**
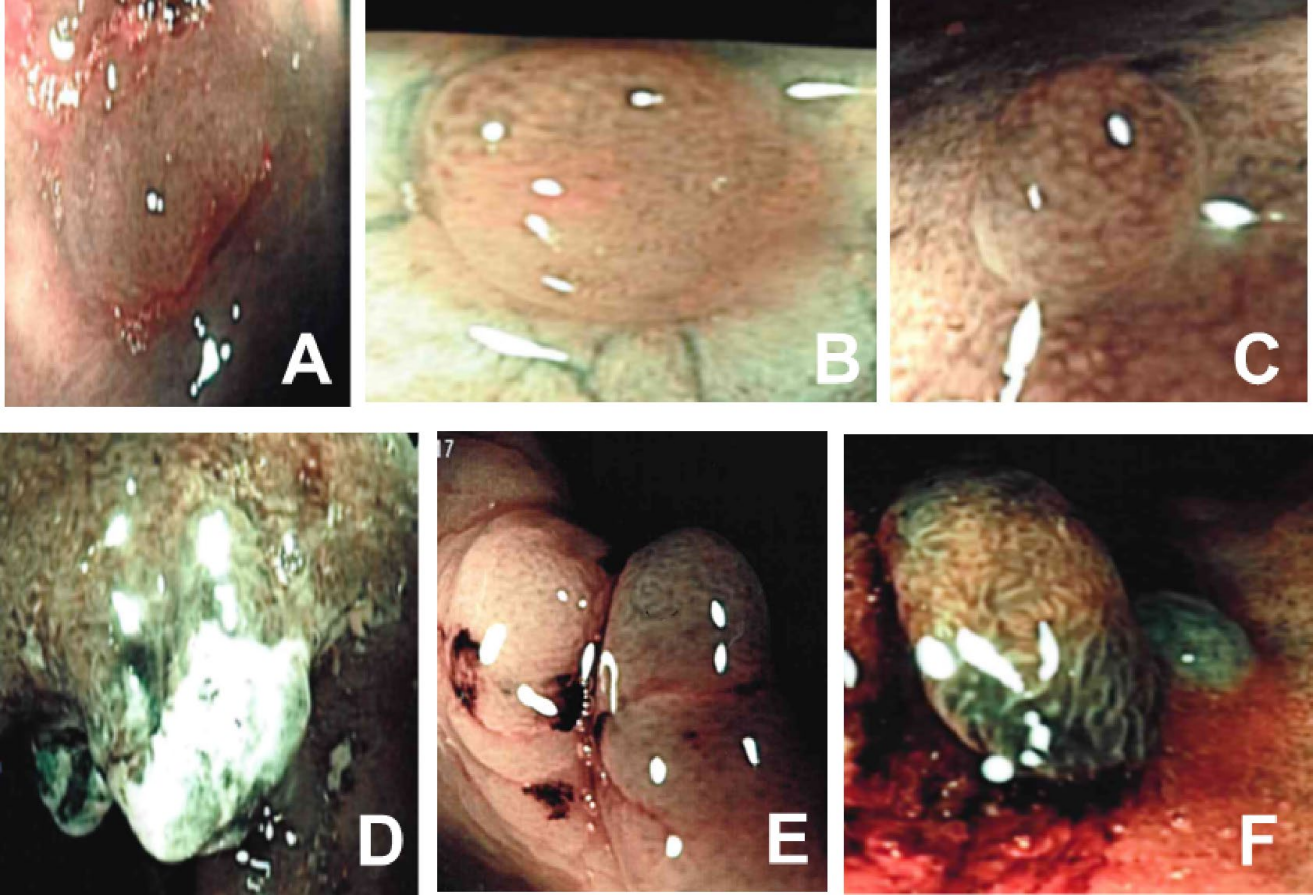
Examples of false negatives (**A**–**C**) and false positives (**D**, **E**) according to the Kudo and/or NICE classifications with NBI. **A** Kudo class II, type-1 NICE (low grade neoplasia). **B** Kudo class I and II, therefore pits-eterogeneity, type-2 NICE (low grade neoplasia). **C** Kudo class II but with pits-eterogeneity, type-2 NICE (low grade neoplasia). **D** Kudo unclassified, focal IIIL-IV with fibrin cap (inflammatory regenerative lesion). **E** Kudo III-L, type-2 NICE (inflammatory polyp). **F** Kudo III-L/IV, type 2 NICE (inflammatory polyp)

Among the three modifiers of the neoplastic risk of Kudo’s classes, intensive vascular pattern and pits heterogeneity were present in three type-I lesions (none neoplastic) and in 21 type-II lesions (3 neoplastic). Therefore, 3 further true positives were correctly recognised, while 3 lesions still were false negatives. On the contrary, fibrin cap was present in 24 previously unclassified lesions, six III-L lesions and two type-IV lesions (none neoplastic), thus decreasing the number of false positives; fibrin cap was never associated to neoplasia. Figure 3 shows some examples of discordance among the three classifications.

The sensitivity, specificity, positive- and negative-likelihood ratios of Kudo-IBD were 86% (95% C.I. 64–97%), 79% (95% C.I. 75–83%), 4.15 (95% C.I. 3.19–5.41) and 0.18 (95% C.I. 0.06–0.51), respectively (Table 3).

### Comparison of classifications

As shown in Table 3, the Kudo-IBD classification had the highest sensitivity, specificity and diagnostic accuracy among the three classifications. Sensitivity of the three methods was not significantly different, while specificity was significantly higher with Kudo-IBD than the other two classifications (*p* = 0.0019 Kudo-IBD vs. Kudo; *p* = 0.0000 Kudo-IBD vs. NICE). Moreover, Kudo-IBD had the best combination in terms of positive and negative likelihood ratios, thus proving to be the most useful and diagnostically accurate test.

## Discussion

In this prospective study, two endoscopic classifications of surface patterns (the Kudo and NICE systems), traditionally used with NBI for optical diagnosis during CRC screening of the general population, have been analyzed in the specific clinical setting of long-standing UC, and compared with a modified Kudo classification which was developed and validated in previous studies with FICE in UC [7, 8].

In our consecutive patients from the real-life, we demonstrate that conventional classifications applied to NBI are not accurate enough in the differential diagnosis between neoplastic and non-neoplastic lesions in UC, mainly because of their low specificity. This is due to the different prevalence of non-neoplastic lesions in UC compared with non-IBD series.

In facts, our sample confirms the higher prevalence of inflammatory lesions compared to neoplastic or hyperplasic lesions in IBD, which has been described in the few studies which included all histological types of visible lesions [4–8]. According to this redundant data from the real world, the criteria for excluding patients with clinical or endoscopic activity, recommended by current guidelines and previously used by many famous studies on DCE in IBD, appears to be unrealistic and does not let us to test the real accuracy of endoscopic criteria for lesions characterization.

On the other hand, the lower accuracy of the two conventional classifications compared with previous reports in non-IBD series [19, 25], should not be surprising given that they were developed in the differentiation between neoplastic and hyperplastic lesions in the screening of general population. Moreover, the high rate of false positives, in both the original Kudo classification and -even more- in the NICE classification, appears plausible as the NICE classification includes dark color as a suspected criteria for neoplasia, which is also expressed by inflammatory lesions in form of hyperemia of the inflamed mucosa [7]; false positives with Kudo classification are most likely given by distortion of the glandular pattern, which is related -by definition- to the mucosal inflammatory infiltrate in IBD.

The SCENIC guidelines were largely silent on pit-pattern classifications because of their inconsistent data in the original chromoendoscopic literature [2], whereas ESGE strongly recommends the use of validated classification systems to support the use of optical diagnosis with advanced endoscopic imaging in the lower GI tract [26]. However, even the most recent guidelines do not define a specific classification accepted for clinical use in IBD [3].

Our modified version of conventional Kudo classification seems to have good accuracy in lesion characterization and can be applied across different technological systems, like FICE and NBI. We believe that our strategy to use the conventional Kudo classification, as the starting platform for stratification of the neoplastic risk, still preserves the biological and clinical significance of the hard work performed in early ’90 by Kudo et al. through their systematic analyses of thousands of lesions [16].

The improved sensitivity of our modified classification is related to the upgrade from low to high-risk lesions for Kudo types I-II in case of two markers, i.e. intensive vascular pattern and pits heterogeneity, which were independent predictive factors of neoplasia in previous studies [27–29]. On the other hand, the improved specificity is related to the use of fibrin cap as a low risk marker of neoplasia, as previously described [7, 22, 30]. Notably, the III-S and V patterns of Kudo classification are still considered markers of very high risk for neoplasia, as well as any other conventional criteria for invasive cancer, like ulcers, depressions, size and strictures, which should be taken in mind despite their absence in our series, in which no invasive cancers have been found.

In our previous experience with FICE, the modified Kudo criteria achieved a sensitivity of 91% and a specificity of 93%, thus achieving current ESGE standards for accurate optical diagnosis of colorectal lesions [31]. In this study with NBI, lower sensitivity (86%) and specificity (79%) were found, with a NPV of 99%. The lower sensitivity than FICE could be related to better image definition of the FICE instrument used with magnification in that past experience. This data suggests the need for studies in which a high-definition and magnification NBI instrument (like the new Evis X1 series) should be used to furtherly test our modified Kudo criteria. Another factor which may have limited the sensitivity of our modified Kudo classification concerns the accuracy of Kudo type II in the absence of other descriptors. In facts, while we confirm that Kudo type I (which describes regular pit pattern identical to those of the surrounding mucosa) was never associated with neoplasia, 6% of our lesions with pit pattern II were neoplastic, similar to the 5% rate found in our previous series with FICE [7]. The risk of false negatives in UC among pit pattern II lesions was previously described using NBI [14, 32], and should be taken in mind, at the expense of lower specificity.

Our study has certainly one major limitation. Colonoscopies were performed by a single operator in a single center, using a new classification which still awaits external validation (which is on going). However, it seems to reply the same clinical results of two previous studies with FICE, which shared similar inclusion criteria [7, 8]. Moreover, no dysplasic serrated lesions were found in our cohort; this is a subgroup of lesions in which the Kudo-IBD criteria, alone or in association with other features, need to be tested and validated.

In conclusion, our study on nearly 400 visible lesions in long-standing UC shows that the diagnostic accuracy of NBI in the optical diagnosis of neoplastic and non-neoplastic lesions is low when used according to the conventional classifications of the non-IBD population, but can be significantly improved by a modified Kudo classification, specific for UC, which already performed well also with FICE. Therefore, the criteria for using NBI in UC surveillance should be re-discussed.

## Data Availability

No datasets were generated or analysed during the current study.
